# Lessons from Outside and Within: Exploring Advancements in Methodology for Naturopathic Medicine Clinical Research

**DOI:** 10.1089/acm.2018.0403

**Published:** 2019-02-22

**Authors:** Janet Schloss, Erica McIntyre, Amie Steel, Ryan Bradley, Joanna Harnett, Rebecca Reid, Jason Hawrelak, Joshua Goldenberg, Claudine Van De Venter, Kieran Cooley

**Affiliations:** ^1^ Australian Research Centre in Complementary and Integrative Medicine, University of Technology Sydney, Faculty of Health, Ultimo, Australia.; ^2^ Office of Research, Endeavour College of Natural Health, Fortitude Valley, Australia.; ^3^ Helfgott Research Institute, National University of Natural Medicine, Portland, OR.; ^4^ Faculty of Pharmacy, University of Sydney, Sydney, Australia.; ^5^ College of Health and Medicine, University of Tasmania, Hobart, Australia.; ^6^ Department of Naturopathy, Bastyr University, Kenmore, WA USA.; ^7^ Faculty of Health Sciences, University of Cape Town, Cape Town, South Africa.; ^8^ Office of Research, Canadian College of Naturopathic Medicine, North York, Canada.

**Keywords:** naturopathy, whole-system research, research methodology, patient centered, pragmatic trials

## Abstract

***Introduction:*** Naturopathy is a mixture of both traditional and complementary medicine. It incorporates a broad set of health care practices that may or may not be traditional to that country or conventional medicine and are not fully integrated into the dominant health care system. Research required to evaluate or substantiate naturopathic medicine may not fall under the testing of randomized clinical trials, which opens up discussions on what is the best practice for research in naturopathic medicine.

***Discussion:*** Not only do advances in health research methodology offer important opportunities to progress naturopathic research, there are also areas where the unique characteristics of naturopathic philosophy and practice can impact other areas of health research. Some of the new advances in health research methodology involve whole-system research, pragmatic trials, template for intervention description and replication protocols for complex interventions, patient-centered care models, and the pragmatic-explanatory continuum indicator summary tool for designing pragmatic trials. Discussion and critique of these health-related methodologies shows that these research methods are more suited for the philosophy and treatment options that naturopathy is based on.

***Conclusions:*** Successful implementation of naturopathic research methodologies, and translation and dissemination of research will require a substantial paradigm shift in which naturopathic practitioners adopt a greater level of responsibility for developing an evidence base for naturopathic medicine.

## Background

Traditional and complementary medicine have a very long history throughout all regions around the world. The World Health Organization (WHO) defines traditional medicine as “the sum total of the knowledge, skill and practices based on the theories, beliefs and experiences indigenous to different cultures, whether explicable or not, used in the maintenance of health as well as in the prevention, diagnosis, improvement or treatment of physical and mental illness.”^1^ Similarly, the WHO defines complementary medicine as “a broad set of health care practices that are not part of that country's own tradition or conventional medicine and are not fully integrated into the dominant health-care system.”^1^ In this way, naturopathy is a system of medicine that aligns with the definitions of both traditional and complementary medicine.

The National Center for Complementary and Integrative Health (USA) defined naturopathy as a whole medical system based on philosophical principles that guide practice and is “classified” as a complementary approach to health care.^2^ Commentators and critics of complementary approaches to health care repeatedly call for quality evidence that is drawn from research that employs randomized controlled trial (RCT) research designs.^3^ This poses several methodological challenges for whole-system interventions such as naturopathy, general practice medicine, chiropractic, acupuncture, and massage. For naturopathy this has been highlighted by The Naturopathic Medical Research Agenda.^4^ Current methodology models such as RCTs are well suited for measuring the effects of chemically defined substances on predefined target tissues in homogeneous well-defined populations with clear attribution to a particular agent. Despite some of the assumptions inherent in this approach, this type of research can be conducted for, by, or on medical, naturopathic, and other complementary medicine professions.^5^ However, a respect for the dynamic interplay between a range of factors that shape health and well-being is inherent to the philosophy of naturopathy and professions that use whole-system interventions and presents tensions, trade-offs, and challenges to effective application of the more conventional RCT design and also begs adoption of other types of research methods appropriate for generating different types of evidence.^6–8^

Within naturopathic practice, interventions are typically tailored for the individual and may involve dietary and lifestyle modifications, mind–body therapies, physical or manual therapies, and ingestible medicines with complex chemical compositions and multiple actions.^4,9,10^ Consequently, research designs must include appropriate measurements that respect and respond to the complexity and various features of naturopathic interventions. The goal of this commentary is to draw attention to key innovations in study design that are relevant to the future of research in the field of naturopathy. Although this article focuses on naturopathy, the principles can be applied to other health professions that use whole-system approaches to health care, including other complementary medicine and conventional medicine professions.^11^ This article explores what naturopathic research and researchers may offer the wider health research community and considers the advancements occurring within health research that will support future robust and rigorous naturopathic research.

## Contemporary Advances in Health Research Methods and Research Tools

Trials involving health care interventions are generally centered around explanatory research utilizing the RCT model—often considered the highest recognized level of clinical evidence.^12^ However, current opinions on explanatory research now recognize that although this type of research may ascertain causal factors (efficacy) in an ideal or controlled situation, it does not confirm whether the intervention is effective in a real-world setting (effectiveness).^13^ To be able to measure effectiveness, pragmatic trials need to be developed and implemented for translational science and application in real-world settings.^12^ The spectrum between explanatory and pragmatic trials is not dichotomous, but can be seen as a continuum with trials incorporating aspects of both in a variety of dimensions.^14^

Certain aspects of naturopathic care can be suited to clinical trials; however, if assessing a holistic intervention, this may not be applicable, due to the perceived reductionist paradigm underpinning the traditional RCT design. Naturopathic medicine is not the only area of health care that has raised this concern. In fact, recognition of the restrictions inherent to the RCT model has launched the preference for pragmatic research designs to evaluate the effectiveness of health care as it really occurs. Most medical and allied health care interventions require an understanding of what is applicable in a real-world setting. The pragmatic-explanatory continuum indicator summary (PRECIS-2) is an instrument that assists researchers in developing trials for this particular purpose.^15^ More importantly, the tool has been useful for articulating important aspects of design and intention, essentially framing what is sometimes a dynamic and disconnected process through the stages of research design, conduct, interpretation, and clinical application.^16^

Being able to combine, develop, and assess trials using the PRECIS-2 model supports researchers to develop trials that provide high-level clinical evidence that allows for individualized clinical decision-making and the delivery of complex multimodal interventions. In addition to the PRECIS-2, a template for intervention description and replication checklist and guide was developed by an international team of experts to assist researchers to promote full and accurate descriptions of trial interventions.^17^ These guidelines are particularly useful for complex interventions, which aligns with the diverse practices inherent to individualized health research.

Focusing on real-world outcomes and effectiveness also increases the need for participatory/community-based involvement.^18^ Equally, an observational or quasi-experimental trial may be advantageous when assessing application in a community-based setting. These types of research designs generally require a mixed-methods approach that is patient centered rather than disease centered.

A focus on designing and implementing patient-centered research has also become more prominent in an era wherein policy makers are emphasizing person-centered care.^19^ Innovation in research methodology has been a necessary response to these policy-driven demands. Fortunately, based on a recognition that clinical outcomes in clinical trials do not capture all important mediators and predictors of real-life clinical practice, several funding agencies including the National Institutes of Health (NIH) and the Patient-Centered Research Outcomes Institute in the United States have endorsed and led the development of research instruments and processes. This includes the Patient Reported Outcomes Measure Information System (PROMIS)^20^ that is used to capture more holistic data on functional, social, emotional, and spiritual domains of health, and more directly involves patients in research.

Overall, the wider health research community has acknowledged the value and importance of a range of diversity in the accepted research designs to answer questions meaningful to the real-world settings and populations. This is evidenced by several studies; for example, an acupuncture pragmatic trial,^21^ an asthma trial being conducted in England,^22^ and a physical activity trial for chronic obstructive pulmonary disease.^23^ These advancements in some way address the concerns underlying previous attempts within complementary medicine research to advocate for whole-system research approaches.^24^

## Adapting and Using Advancements in Health Research to Examine Naturopathic Medicine

The diversification of accepted health research methods to include pragmatic designs supporting assessment of complex patient-centered interventions, as outlined in the previous section, provides important opportunities for researchers focusing on the real-world practice of naturopathic medicine. This type of research is important because patients who require a variety of different interventions due to complex disease status are not normally included in certain trials since they do not fit the “optimal” requirements for that trial (e.g., too many potentially confounding health complications). In pragmatic trials, all patients who have the conditions of interest, regardless of their responsiveness, past compliance, and comorbidities, can be enrolled.^25^

Recent research evaluating treatments for low-back pain provides excellent examples of multiple research methodologies highly applicable to naturopathic practice, and practices, including inclusion of education and self-care practices in randomized trials^26–29^; development and inclusion of multidimensional patient-reported outcome measures^30–32^; application of mixed-method designs to capture patients' experiences with the intervention^33,34^; evaluating assessments of individual predictors of outcomes^35,36^ including experience of care,^37,38^ inclusion of informed choice,^39,40^ and expectations^41^ as predictive factors for improved clinical outcomes. These research methodologies are richly aligned with naturopathic principles of “doctor as teacher” (*Docere*), “hierarchy of therapeutics,” and “treat the whole person” (*Tolle Totum*)^42^ because there is significant patient engagement, attention to education and self-reflection, as well as assessing aspects of the whole person as part of the intervention or outcome.

Several of the methods described have been applied to clinical research evaluating naturopathic practice. For example, studies in primary prevention of heart disease collecting data on the outcomes of highest priority to patients in addition to traditional Framingham risk scores,^43^ and quasi-experimental research in type 2 diabetes collecting patient-reported outcomes including self-efficacy and stress, in addition to clinical hemoglobin A1c changes,^44^ and including qualitative elements to capture patients' experiences with care.^45^

Other research has also been published that describes patients experiencing person-centered care when treated by a naturopathic medicine clinician.^46^ For this reason, the patient-centered research methods being developed within the broader health research community have particular relevance within naturopathic research. In fact, instruments such as PROMIS and other patient-reported outcome measures afford researchers an opportunity to capture changes to health status as experienced by the patients themselves.

The nature of naturopathic practice and the practices of most clinical disciplines are complex to research in totality. However, the pragmatic and patient-centered research methods emerging from innovations in health research methods provide an approach to interrogate the complexity of practice while not requiring violation of fundamental naturopathic principles of practice, allowing high external validity in the study design. In fact, these new research methods may help determine fidelity to complex naturopathic practices previously undervalued or overlooked in health research.^47^

## The Potential Contribution of Naturopathic Approaches and Perspectives to Strengthen Health Research

Not only do advances in health research methodology offer important opportunities to progress naturopathic research and benefit patients, there are also areas where the unique characteristics of naturopathic philosophy and practice can impact on other areas of health research. The *tolle totem* principle of naturopathy that focuses on treatment of all aspects of the individual requires clinicians to acknowledge the complexity of disease etiology and pathophysiology.^48,49^ In doing so, naturopathic clinical understanding may open up new avenues for researchers from other disciplines to explore. A recent example of this is the growing research interest in the clinical importance of gastrointestinal health in an array of health conditions^50–54^—a concept well established within the naturopathic clinical approach.^55^ There are undoubtedly many other areas where the insights and experience of naturopathic clinicians may, once communicated to a wider audience through case reports and medical hypothesis articles, encourage more research breakthroughs that will benefit the community in ways as yet unmeasured.

Such an opportunity to capture clinical insights as a basis for future research may not only assist the substantive topic in question, it may also offer a practical method for recalibrating the balance within the evidence-based medicine triad in general, serving to bolster the value and awareness of the pillars of clinical expertise and patient values through research.^56–58^ As the naturopathic profession works with researchers to document and share the experience and insights of clinicians (both past and present), naturopaths and researchers will provide a model through which the “clinician experience” pillar of evidence-based medicine can be operationalized.^58^

Naturopaths are well placed to support new research by effectively and rapidly implementing practices developed through new areas of research such as precision or personalized medicine,^59^ thereby providing opportunities to better understand the real-world implications of the health technology as it develops. In fact, the emphasis on individualized treatments as a core philosophical element of naturopathic care^55^ may mean that naturopaths are more ideologically and logistically prepared to incorporate such personalized health care than conventional health professionals. However, despite a natural and opportune fit, issues with capacity, mentorship, and training and support for naturopath scientists and cross-disciplinary teams need to be addressed.^60,61^

There are still gaps in the available health research methods and instruments, which limits the robustness of some facets of naturopathic research. The authors cannot meaningfully build the experience and knowledge of past (i.e., historical) naturopaths into the design of research projects, for example, without a rigorous framework to guide the analysis and appropriate use of traditional information sources (e.g., historical texts and ancestral or elder-based knowledge).^62^ The authors also need to develop instruments that measure the outcomes uniquely important in naturopathic clinical decision-making and treatment evaluation (e.g., vital force). In some instances, some relevant instruments may already exist that only require small modifications to capture nuances specific to naturopathic principles and practice. In other cases, the instruments will need to be developed in full.

## Implications and Future Directions

There are several implications related to naturopathic medicine research methodology that require careful consideration. One major challenge to conducting naturopathic research is the need for adequate infrastructure, which includes practitioner research capacity, consumer and practitioner engagement, and integration into health care systems, all of which are not fully developed in naturopathic medicine. The lack of integration of naturopathic health services in health care systems prevents access to resources to assist clinical research, such as health databases (e.g., e-health records) and practitioner databases (e.g., registration agencies). Creating practice-based research networks (PBRNs) or academic networks^63^ are potential solutions that enable researchers to access practitioners and their patients.^64^

PBRNs will also help facilitate a research culture within naturopathy by providing an opportunity for practitioners to participate in research within grass roots practice.^65^ Lack of clinician research capacity is a barrier to conducting research such as pragmatic trials, translating research, codifying knowledge, and developing suitable research methods. Naturopathic practitioners in some situations are adequately trained to adopt a researcher–practitioner model of practice in which research and clinical skills are equally valued. To enable naturopathic practitioners to be involved in the research process, there needs to be adequate educational infrastructure to increase research capacity. There is currently insufficient undergraduate, graduate, and postgraduate education in health and social science research methods in naturopathic medicine.^66^ This shortfall needs to be urgently addressed, otherwise lack of research skills will continue to be a significant barrier for practitioners to participate in and translate naturopathic research.

There is an enormous amount of research needed in naturopathic medicine. Participatory community-based methods such as Delphi techniques could be used to engage naturopaths and consumers to determine naturopathic research priorities.^67,68^ It is critical that naturopathic research is translatable to clinical practice and meaningful to health care consumers. Delphi techniques allow for clinician participation in design of the research process to ensure clinically meaningful outcomes and provide an opportunity to involve health consumers in research to ensure it is patient centered. Consensus methods such as Delphi would also be suitable for identifying methods for research translation to both naturopathic practice and health care consumers. This participatory approach could be extended to assist with codifying knowledge, which includes developing clinical guidelines for naturopathic care. These methods could also facilitate the consolidation of traditional evidence into meaningful frameworks that are accessible to clinicians and the public. An example of this is described in an article that discusses the naturopathic approaches to irritable bowel syndrome.^69^

Developing and evolving naturopathic research methodologies can be considered an iterative process that has the potential to influence health research more broadly. However, the advancements in health research methodologies more generally afford an opportunity for naturopathic research to align with established research designs while still answering clinically relevant and philosophically sensitive research questions.

However, successful implementation of naturopathic research methodologies, and translation and dissemination of research will require a substantial paradigm shift in which naturopathic practitioners adopt a greater level of responsibility for developing an evidence base for naturopathic medicine. Initiatives to support and evaluate knowledge mobilization^70^ within the community of naturopathic medical research, education, and practice may play a key, but yet unexplored role.^71^ Researchers in this field have an important leadership role to effectively facilitate this transformation, which will benefit health consumers, naturopathic practitioners, and health care systems they serve. Practitioners who are not in the research field can also contribute by being part of PBRNs, therefore, assisting in this paradigm shift and allowing the leaders in the field to move forward.
